# Human lung telocytes could promote the proliferation and angiogenesis of human pulmonary microvascular endothelial cells in vitro

**DOI:** 10.1186/2052-8426-2-3

**Published:** 2014-02-01

**Authors:** Yonghua Zheng, Xiaoke Chen, Mengjia Qian, Miaomiao Zhang, Ding Zhang, Chunxue Bai, Qun Wang, Xiangdong Wang

**Affiliations:** Department of Respiratory Medicine, Shanghai Respiratory Research Institute, Zhongshan Hospital, Fudan University School of Medicine, Fenglin Rd, No 180, Xuhui Dist, Shanghai, 320003 China; Department of thoracic surgery, Shanghai Respiratory Research Institute, Zhongshan Hospital, Fudan University School of Medicine, Fenglin Rd, No 180, Xuhui Dist, Shanghai, 320003 China; Biomedical Research Center, Shanghai Respiratory Research Institute, Zhongshan Hospital, Fudan University School of Medicine, Fenglin Rd, No 180, Xuhui Dist, Shanghai, 320003 China

**Keywords:** Human lung, Telocytes, VEGF, EGF, Proliferation, Angiogenesis

## Abstract

**Background:**

In the previous studies, telocytes were found near the capillaries in many tissues, especially on the extracellular matrix of blood vessels and positive to CD34 and c-kit. Therefore, the present study aimed to explore if telocytes could produce angiogenesis associated cytokines, promote the proliferation and the angiogenesis of vascular endothelial cells in vitro.

**Methods:**

Human lung telocytes were isolated and cultured, and were identified by immunofluorescence cytochemistry with CD34, c-kit and vimentin. Telocytes conditional media (TCM) was prepared, and the expressions of angiogenesis associated cytokines in TCM were detected by ELISA. Human pulmonary microvascular endothelial cells (HPMECs) were cultured with DMEM media or TCM for 72 hours. The proliferation of HPMECs was continuously detected with CCK-8 kit at an interval of 12 hours. HPMECs were also injured by lipopolysaccharide, and cultured with TCM and DMEM respectively, and the tube formation capacity was detected.

**Results:**

Telocytes were positive for CD34, c-kit and vimentin. The expression**s** of VEGF and EGF in TCM were significantly higher, the proliferation of HPMECs cultured with TCM significantly increased, and the tube formation of HPMECs injured by endotoxin was improved with the culture of TCM, as compared with the culture of DMEM.

**Conclusion:**

The present study provides the evidence that human lung telocytes could produce the growth factors, such as VEGF and EGF. Telocytes conditional media induced the proliferation of pulmonary endothelial cells and prevented from endotoxin-induced compromise of pulmonary endothelial angiogenesis.

## Background

Telcoytes as a new type of interstitial cells are characterized by the extensive cellular body with elongation and distinguished from other cells by telopodes, the long, thin and miniliform prolongations [1, 2], which could only be observed under electron microscope in the vital tissues [3]. Telocytes were suggested to be positive for CD34, c-kit and vimentin [2] and found mainly in the interstitial space of multiple organs and tissues in mammals, such as heart [1, 4, 5], lung [6–8], intestine [9], uterus and fallopian tube [10], urinary bladder [11], skeletal muscle [12, 13], pancreas [14], parotid glands [15], meninges and choroid plexus [16], placenta [17] and skin [18, 19].

Telocytes were identified to be located on the extracellular matrix of blood vessels, e.g.arterioles, venules and capillaries [20], and connected with capillaries in the heart [21]. Our previous studies demonstrated that telocytes were situated in the interstitial space of the lung tissues between the smooth muscular cells and blood capillary endothelia cells [6, 7, 22]. It was proposed that telocytes might participate in the structure of air-blood barrier. The number of telocytes significantly increased around the neocapillaries in the heart of mice with acute myocardial infarction [23], indicating that telocytes may contribute to the angiogenesis of blood capillaries. The present study aims to explore if telocytes could secrete angiogenesis associated factors and promote the proliferation and the angiogenesis of vascular endothelial cells in vitro.

## Methods

Human lung samples were obtained from the patients undergoing surgery for lung cancer, of which the normal tissue was defined as the location 15 cm from the tumor tissue and proved by light microscope. The application of human tissue for research was approved by the Ethical Evaluation Committee of Zhongshan Hospital, Fudan University, Shanghai, China. Human lung telocytes were isolated and cultured with the methods that were reported previously [6, 24]. Telocytes were cultured in Dulbecco’s Modified Eagle’s Medium (DMEM, Gibco, USA) with high glucose-complete medium, including 10% fetal bovine serum(GIBCO, USA), 100 UI/ml penicillin, and 0.1 mg/ml streptomycin (Sigma Chemical, USA). Human pulmonary microvascular endothelial cells (HPMECs) were purchased from ScienCell Research Laboratories (Catalog 3000, Lot 3395, CA, USA) and cultured in PBS.

### Morphology of human lung telocytes

Telocytes were examined and photographed by phase contrast microscope, under an inverted phase contrast microscope (Olympus 1 × 51, Japan). For staining with Mito Tracker Green FM, telocytes were incubated in phenol red-free DMEM with 10% fetal bovine serum and labeled with 80 nM Mito Tracker Green FM (Beyotime C1048-50 μg, China), incubated at 37°C for 30 min in a humidified atmosphere, 5% CO_2_ in the air, subsequently washed with DMEM medium, and examined and photographed by fluorescence microscopy with 450–490 nm excitation light, 520 nm barrier filter (Olympus 1 × 51, Japan).

### Immunofluorescence cytochemistry

Cultured telocytes were washed with PBS and fixed by acetone for 5 min, then rinsed with PBS and incubated with 0.5% Triton X-100 for 30 min at room temperature. Cells were blocked by using goat serum for 1 h at 37°C, after buffer was removed. Immunostaining was performed with goat anti-vimentin (abcam, ab11256, USA), mouse anti-CD34 (abcam, ab6330, USA), rabbit anti-c-kit (abcam, ab5506, USA). Three primary antibodies were diluted in PBS at a dilution of 1:100 for vimentin, 1:200 for CD34 and 1:100 for c-kit and incubated for 6 hrs at 4°C. Telocytes were washed and incubated with secondary antibodies: blue goat anti-rabbit (Beyotime, DyLight405, China), blue goat anti-mouse (Beyotime, A0412, China) and red donkey anti-goat (Beyotime, A0502, China) at a dilution of 1:500 for 30 min at 37°C. Cells were then washed and counterstained with DAPI. Stained cells were observed and photographed by using a fluorescence microscope through 40× objective (Olympus 1 × 51, Japan).

### Dynamic real-time observations

Dynamic movement of telocytes were measured by the real-time cell monitoring system, using a Cell-IQ cell culturing platform (Chip-Man Technologies, Tampere, Finland), equipped with a phase-contrast microscope (Nikon CFI Achromat phase contrast objective with 10 magnification) and a camera. The equipment was controlled by Imagen software (Chip-Man Technologies). Three or four visual fields were automatically selected in each well, of which images were captured and autographed at 20 min intervals for 96 h.

### RNA isolation and preparation

Human lung telocytes were cultured and collected at 0, 24, or 48 hours, respectively, for RNA preparation by using TRIzol reagent (Invitrogen life technologies, Carlsbad, CA) and the RNeasy kit (Qiagen, Valencia, CA) according to the manufacturer’s instructions, including a DNase digestion treatment. The amount and quality of RNA was measured on NanoDrop-1000 spectrophotometer and monitored with the Agilent 2100 Bioanalyzer (Agilent Technologies, Santa Clara, CA). RNA integrity was assessed by standard denaturing agarose gel electrophoresis.

### Angiogenesis

Human lung telocytes were planted in two 6-well culture plates (1 × 10^6^/well) and cultured with DMEM without fetal bovine serum (2.5 ml/well) for 24 and48 h, respectively. The supernatant was collected, filtered through a 0.22 μm membrane, and stored at -80°C for further usage. The supernatants were analyzed for the expressions of angiogenesis associated cytokines with corresponding ELISA kits, and the DMEM without fetal bovine serum was chosen as controls. A total of 8 factors and cytokines were detected, including vascular endothelial growth factor (VEGF), endothelial growth factor (EGF), granulocyte macrophage colony-stimulating factor (GM-CSF), tumor necrosis factor-α (TNF-α), interferon-γ (IFN-γ), monocyte chemoattractant protein-1(MCP-1), tissue inhibitor of matrix metalloproteinase-1 (TIMP-1), and tissue inhibitor of matrix metalloproteinase-2 (TIMP-2). All ELISA kits were taken from eBioscience (California, USA) and the procedures were carried out according to the manufacturer’s instructions.

### Proliferation of HPMECs

The proliferation of HPMECs was detected by cell counting kit-8 (CCK-8) (Dojindo Laboratories, Kumamoto, Japan). Briefly, HPMECs were cultured in 96-well plates (1 × 10^4^/well) for 48 hours, and divided into 1) HPMECs were cultured in PBS with or without telocytes, 2) HPMECs were cultured in PBS with or without VEGF, or 3) HPMECs were co-cultured with telocytes with or without mono-antibody against VEGF, respectively. After the treatment, 10 μl of CCK-8 solution was added to each well at each time point, and the 96-well plate was continuously incubated at 37°C for 1 h. The value of optimal density for each well was measured at wavelength 450 nm to determine the cell proliferation on a microplate reader (Multiskan, Thermo, USA).

### Tube formation assay of HPMECs

The tube formation capacity of HPMECs was detected with Matrigel (Cat 356234, BD, USA). Briefly, Matrigel at 100 μl was paved evenly in 96-well culture plates, and were incubated at 37°C for 1 h until the gel became coagulated. HPMECs in logarithmic growth phase were put into 96-well plates (2 × 10^4^/well) paved with Matrigel. Cells were cultured in DMEM and stimulated with vehicle or lipopolysaccharide (LPS) at 2 or 10 μg/ml, or in TCM and stimulated with vehicle or lipopolysaccharide (LPS) at 2 or 10 μg/ml, respectively. Tube formation of HPMECs was observed under phase contrast microscope (Olympus 1 × 51, Japan).

### Statistical analysis

Data were represented as means ± SD and experimental differences were tested for statistical significance by using analysis of variance and paired t-test. *P* values less than 0.05 were regarded as statistically significant.

## Results

**Figure 1:**
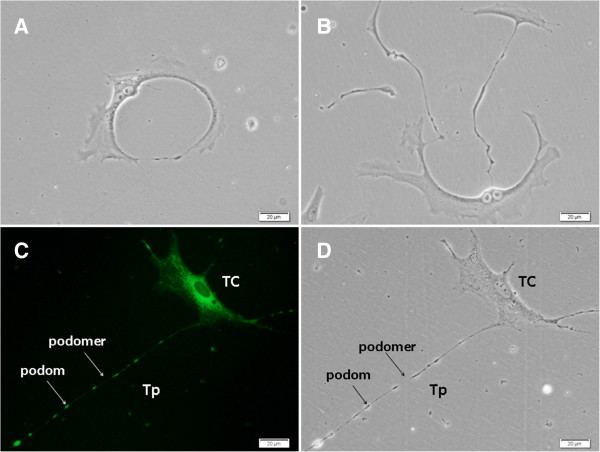
**Culture of human lung telocytes (TCs).** As shows in **A**, **B** and **C**, TCs have long and flat cellular body with longer telopode (Tp), and there are many granular materials both in their cellular body and Tp. Tps with divarications and they connect together. **D** showed that the mitochondria mainly located within TCs body and podoms (arrows), which was observed through fluorescence microscope. Cells were observed by phase-contrast microscopy, and the magnification was 400 × .

**Figure 2:**
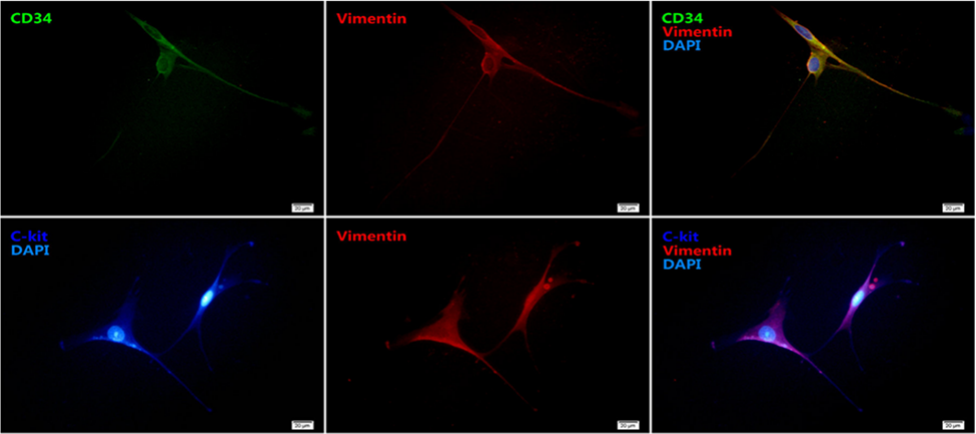
**Telocytes were immunostained for CD34 (green), c-kit (dark blue), vimentin (red) and DAPI (blue) the magnification is 400×, and the scale bar is 20 μm.**

**Figure 3:**
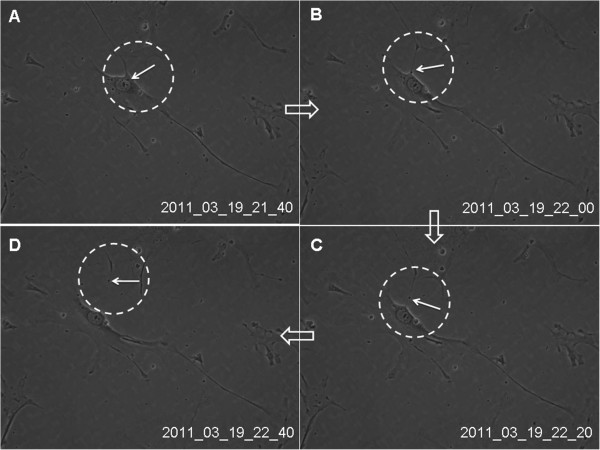
**Real-time dynamic observation of cultured lung telocytes through cell IQ cell culturing platform.** The granular material (black arrow in the dotted black circle) moved around from telocytes body to telopodes, and excreted outside the cell **(A-D)**. Images were captured at 20 min intervals. Magnification is 200 × .

**Figure 4:**
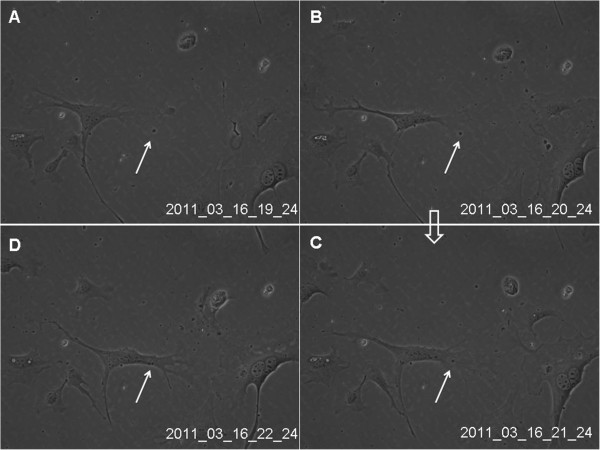
**Real-time dynamic observation of cultured lung telocytes through cell IQ cell culturing platform.** The granular material (black arrow) appeared within the cellular frame, attracted cytoplasma, and was rounded with rich cytoplasma **(A-D)**. Images were captured at 20 min intervals. Magnification is 200×. HPMECs were divided into two groups and cultured for 72 hours with DMEM and TCM respectively, and the proliferation of the cell was detected at 0 h, 12 h, 24 h, 48 h and 72 h. As showed in the figure, the proliferation of HPMECs cultured with TCM was faster than that of DMEM (*P* < 0.05).

Human lung telocytes were identified with clear telopodes, podoms, or podomers 3–5 days after the primary culture (Figure 1A). The length was related with the duration of culture in the early phage of cell growth. Telopodes could connect with each other frequently by either side-to-side or end-to-end of telopodes and occasionally by end-to-side (Figure 1B). Mitochondria were identified in telocytes body and telopode stained with or without *Mito Tracker Green* (Figure 1C and D). The results from immunofluorescence cytochemistry showed that telocytes were positive for CD34, c-kit and vimentin (Figure 2). Through the real-time observation from Cell IQ cell culturing platform, those granular materials moved around from cellular body to telopodes, excrete outside the cell (Figure 3A-D) and were captured by other telocytes (Figure 4A-D).

**Figure 5:**
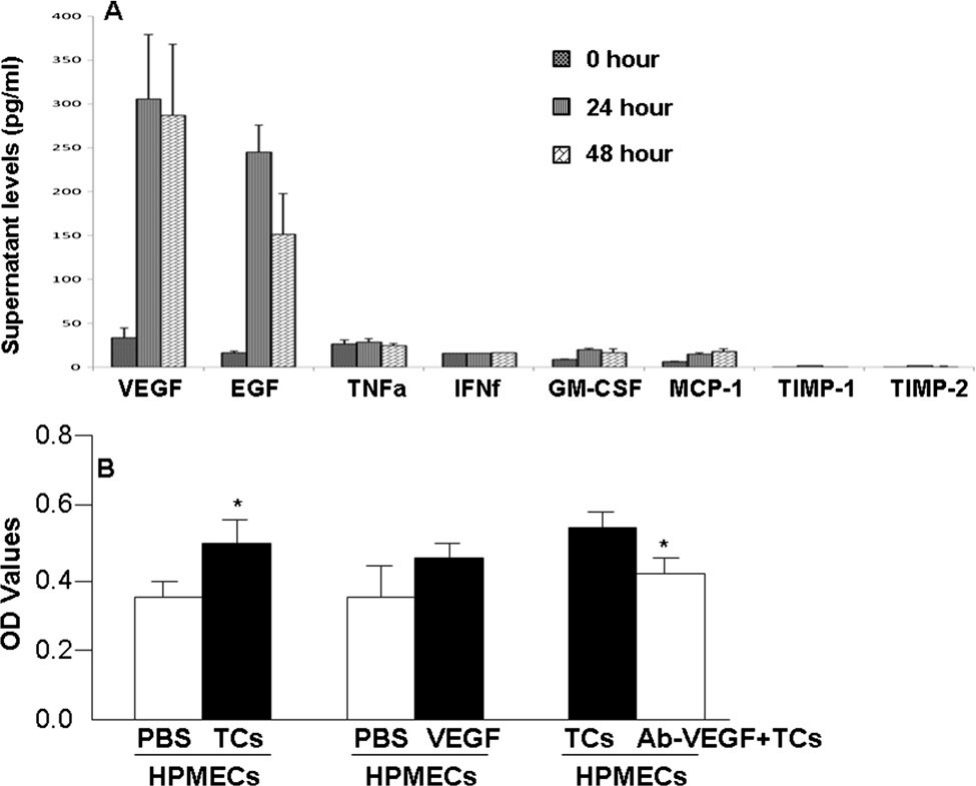
**Supernatant levels of different inflammatory mediators at 0, 24, or 48 hours of telocyte culture.** There were significantly increased levels of VEGF and EGF **(A)**. The proliferation rate of HPMECs was significantly increased after the co-culture with telocytes, as compared with those with PBS (*P*< 0.05, **B**). VEGF per se did not increase the proliferation of HPMECs significantly, while telocytes treated with mono-antibody against VEGF could significantly reduce the proliferation of HPMECs (*P*< 0.05, **B**).

Levels of angiogenesis associated cytokines in the supernatant of telocytes and DMEM (without fetal bovine serum) were detected respectively, by using corresponding ELISA kits. Levels of VEGF and EGF in the telocytes supernatant were significantly higher than in controls (*P* < 0.05), as shown in Figure 5A. There is no difference in levels of GM-CSF, TNF-α, IFN-γ and MCP-1 between telocytes supernatant and DMEM. Figure 5B demonstrated that the proliferation rate of HPMECs significantly increased after the co-culture with telocytes, as compared with those with PBS (*P* < 0.05, Figure 5B). VEGF per se did not increase the proliferation of HPMECs significantly, while telocytes treated with mono-antibody against VEGF could significantly reduce the proliferation of HPMECs (*P* < 0.05, Figure 5B).

**Figure 6:**
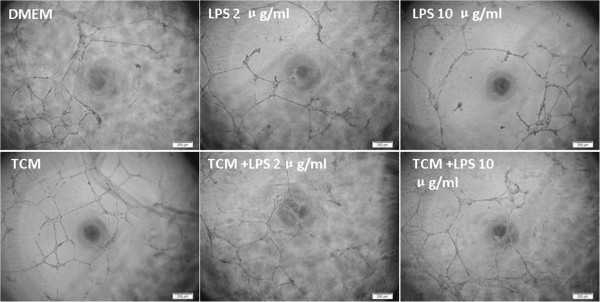
**The tube formation assay of HPMECs.** The ability of tube formation in HPMECs cultured with TCM increased significantly, as compared with DMEM. The tube formation of HPMECs was damaged after the stimulation with different dosages of LPS. DMEM complete medium could not recover LPS- induced damage, while the damage could be partially recovered through the culture with TCM. The magnification is 100×, and the scale bar is 200 μm.

The tube formation assay of HPMECs. Being compared with DMEM, the ability of tube formation in HPMECs cultured with TCM was increased significantly. After the stimulation with different dosages of LPS, the tube formation of HPMECs was damaged, and DMEM could not recover this damage, while the damage was partially recovered through the culture with TCM (Figure 6).

## Discussion

There are increasing histological and morphological studies reveal that telocytes exist in almost all organs and tissues in mammals, but there is still a short of the information on the function of telocytes correspondent to their characteristics in the tissues. Popescu et al. [8] found that lung telocytes were connected with stem cells and proposed a nutritional role of telocytes for lung tissue stem cells. Lung telocytes may play a role during the process of regeneration and reparation for the alveolar and bronchial epithelial cells, airway smooth muscle, or pulmonary microvascular endothelial cells, evidenced by the previous finding that telocytes could support the tissue re-organization in development of adult heart [25]. Our previous studies described that lung telocytes were mainly distributed in the alveolar interstitial connected tightly with alveolar epithelia cells and participated in the structure of air-blood barrier, in the small vein and bronchioles and in the interstitial space of smooth muscle participated in the frame structure of the blood and bronchioles [6, 7, 22]. Telocytes are positive to CD34 and C-kit which expressed on the surface of hemopoietic stem cells, and are proposed to participate in the angiogenesis.

The angiogenesis is a common phenomenon both in physiological and pathological conditions, including proliferation of vascular endothelial cells, enzymatic degradation of basement membrane and interstitial matrices by endothelial cells, migration of vascular endothelial cells, or eventually formation of a blood vessel tube from sprouting vascular endothelial cells. Angiogenesis could be induced by activation of VEGF and EGF receptors by the binding of VEGF and EGF to induce the tube formation in the vascular endothelial cells, promote endothelial cell proliferation and vascular permeability, and maintain newly-formed blood vessels [26, 27].

The present study described that production of VEGF and EGF from human lung telocytes increased significantly. Cultured medium of telocytes could promote the proliferation of HPMECs, and partially recover the ability of tube formation of HPMECs injured by LPS. Our data indicate that telocytes may participate in the angiogenesis of lung tissues, in both normal and pathological conditions and play roles as progenitor cells and nutrient cells during the regeneration and reparation of the injured tissues. Telocytes can be a new therapeutic target in lung diseases.

## Conclusion

The present study provides the evidence that human lung telocytes could produce the growth factors, such as VEGF and EGF. Conditional culture medium of those telocytes induced the proliferation of human pulmonary microvascular endothelial cells and prevented from endotoxin-induced compromise of pulmonary endothelial angiogenesis. Human pulmonary telocytes might play an important role during the process of angiogenesis of endothelial vascular cells.
